# Smoking prevalence and nicotine dependence among teachers

**DOI:** 10.1192/j.eurpsy.2026.10814

**Published:** 2026-07-31

**Authors:** I. Sellami, A. Abbes, A. Feki, D. Elleuch, M. L. Masmoudi, M. Hajjaji, K. Jmal Hammami

**Affiliations:** 1 Occupational Medicine; 2 Rheumatoloy, Hedi Chaker Hospital; 3 Family Department, Medecine University, Sfax, Tunisia

## Abstract

**Introduction:**

As role models for young people and key players in prevention in schools, teachers occupy a strategic position in the fight against smoking. Therefore, the prevalence of smoking among primary and secondary school teachers deserves special attention.

**Objectives:**

Our study aimed to explore the smoking behaviour among teachers.

**Methods:**

We conducted a descriptive and analytical cross-sectional study among teachers working in public schools in the Sfax region in Tunisia, during October, November and December 2016. We used a questionnaire covering socio-demographic characteristics, professional data and smoking behaviour. We assessed the level of nicotine dependence using the Fagerström test.

**Results:**

We included 485 teachers working in public schools in the Sfax region of Tunisia. The mean job tenure was 18.83 ± 10.92 years. The prevalence of smoking was highest among teachers aged 20–29 years (21.4%), followed by those aged 40–49 years (19.2%). The mean nicotine dependence score was 5.48 ± 2.67. Among all current smokers, 56% had nicotine dependence classified as severe to very severe. Statistical analysis did not reveal a significant association between smoking status and age (p = 0.794) or job tenure (p = 0.102).

**Conclusions:**

Smoking prevalence among teachers in Sfax was moderate, yet more than half of current smokers exhibited severe nicotine dependence, highlighting a critical health concern.

**Disclosure of Interest:**

None Declared

